# Revisiting epithelial‐mesenchymal transition in cancer metastasis: the connection between epithelial plasticity and stemness

**DOI:** 10.1002/1878-0261.12096

**Published:** 2017-06-26

**Authors:** Tsai‐Tsen Liao, Muh‐Hwa Yang

**Affiliations:** ^1^ Institute of Clinical Medicine National Yang‐Ming University Taipei Taiwan; ^2^ Genome Research Center National Yang‐Ming University Taipei Taiwan; ^3^ Division of Medical Oncology Department of Oncology Taipei Veterans General Hospital Taiwan

**Keywords:** epithelial‐mesenchymal transition, metastasis, plasticity, stemness

## Abstract

Epithelial‐mesenchymal transition (EMT) is an important process in embryonic development, fibrosis, and cancer metastasis. During cancer progression, the activation of EMT permits cancer cells to acquire migratory, invasive, and stem‐like properties. A growing body of evidence supports the critical link between EMT and cancer stemness. However, contradictory results have indicated that the inhibition of EMT also promotes cancer stemness, and that mesenchymal‐epithelial transition, the reverse process of EMT, is associated with the tumor‐initiating ability required for metastatic colonization. The concept of ‘intermediate‐state EMT’ provides a possible explanation for this conflicting evidence. In addition, recent studies have indicated that the appearance of ‘hybrid’ epithelial‐mesenchymal cells is favorable for the establishment of metastasis. In summary, dynamic changes or plasticity between the epithelial and the mesenchymal states rather than a fixed phenotype is more likely to occur in tumors in the clinical setting. Further studies aimed at validating and consolidating the concept of intermediate‐state EMT and hybrid tumors are needed for the establishment of a comprehensive profile of cancer metastasis.

**Abbreviations**
CBP: CREB binding protein
CSCs: cancer stem cells
CtBP1: C‐terminal binding protein 1
EMT: epithelial‐mesenchymal transition
EMT‐TF: epithelial‐mesenchymal transition transcription factor
ERCC1: ERCC excision repair 1, endonuclease noncatalytic subunit
HDAC: histone deacetylase
HIF‐1α: hypoxia‐inducible factor 1 alpha
Id1: inhibitor of differentiation 1
IL‐8: interleukin‐8
LSD1: lysine‐specific demethylase
MET: mesenchymal‐epithelial transition
OVOL2: ovo‐like zinc finger 2
PRC2: polycomb repressive complex 2
Snail1: Snail family zinc finger 1
TGF‐β: transforming growth factor beta
Twist1: twist family bHLH transcription factor 1
ZEB1: zinc finger E‐box binding homeobox 1
ZEB2: zinc finger E‐box binding homeobox 2

## 1 General overview of EMT

**Figure 1:**
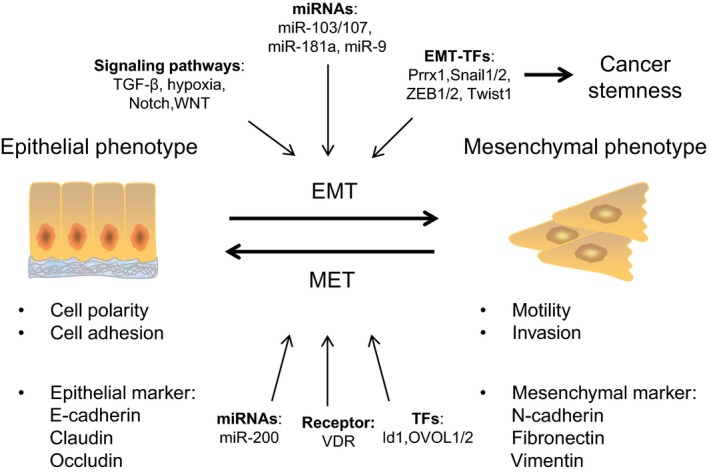
The dynamic change between the epithelial and the mesenchymal phenotype in cancer cells during metastasis. In response to EMT‐triggering events, such as the activation of signaling pathways (e.g., TGF‐β, hypoxia, Notch, WNT) or the expression of EMT‐TFs (e.g., Snail1/2, Twist1, ZEB1/2, Prrx1) and miRNAs (e.g., miR‐103/107, miR‐181a, miR‐9), cancer cells transition from an epithelial phenotype to a mesenchymal phenotype, with the suppression of epithelial markers and expression of mesenchymal markers. Activation of an EMT program results in the acquisition of migration and invasion abilities for facilitating cancer dissemination. Furthermore, EMT‐TFs promote cancer cells to acquire the stem‐like features. After the mesenchymal‐type cancer cells reaching the metastatic sites, the cancer cells reverse back to the epithelial type through MET, which is critical for cancer colonization. The effectors of MET include the activation of certain transcriptional factors (e.g., Id1, OVOL1/2), miRNAs (e.g., miR‐200), and receptor (VDR).

During embryonic development, epithelial cells lose their polarity and are converted into a mesenchymal phenotype. This process is referred to as epithelial‐mesenchymal transition (EMT) (Nieto *et al*., 2016). The classic view of EMT is that epithelial cells transform into mesenchymal cells. Morphological changes in cells have been considered the characteristic feature of EMT (Hay, 1995; Nieto, 2013). EMT presents certain features that are considered as its hallmarks, including disruption of intercellular junctions, loss of cell polarity, reorganization of the cytoskeleton, and increased cell motility. Therefore, in most experimental models, epithelial (E‐cadherin) and mesenchymal (N‐cadherin and vimentin) markers and morphological changes are examined as indicators to confirm the occurrence of EMT. In cancers, EMT is triggered by diverse signaling pathways through the regulation of EMT transcription factors (EMT‐TFs) and/or microRNAs (miRNAs) (Nieto *et al*., 2016). EMT not only enhances cancer motility and dissemination through the disruption of intercellular junctions but also allows cells to acquire stem‐like properties (Nieto *et al*., 2016). However, the reverse process of EMT, that is, mesenchymal‐epithelial transition (MET), is an important process for cancer cell re‐differentiation and metastatic colonization (Bonnomet *et al*., 2012). Therefore, the association between EMT‐MET and stemness is controversial and debated. The major factors and signaling pathways that trigger the changes in EMT/MET are summarized in Fig. 1. In this review, we summarize and discuss the connection between epithelial and mesenchymal states and the acquisition of stemness in cancer cells.

### 1.1 EMT transcription factors

One of the major events contributing to EMT is the activation of EMT‐TFs, such as Snail1, Twist1, ZEB1, and ZEB2. These EMT‐TFs often control the expression of each other and cooperate with other TFs to regulate the expression of target genes, and EMT‐TFs often function as repressors for epithelial genes and activators for mesenchymal genes (De Craene and Berx, 2013; Peinado *et al*., 2007).

#### 1.1.1 Snail1

Snail1 (also known as Snail) functions as a suppressor by binding to the E‐box in the promoters of the junction proteins E‐cadherin, claudin, and occludin and recruiting histone modifiers, including SIN3A‐histone deacetylase 1 and 2 (HDAC1 and HDAC2) complex, polycomb repressive complex 2 (PRC2), and lysine‐specific demethylase 1, to repress the transcription of target genes (Batlle *et al*., 2000; Cano *et al*., 2000; Herranz *et al*., 2008; Ikenouchi *et al*., 2003; Lin *et al*., 2010a,b; Peinado *et al*., 2004). However, Snail1 also acts as an activator that increases the expression of mesenchymal genes such as fibronectin 1, an extracellular matrix protein (Stanisavljevic *et al*., 2011); excision repair 1 endonuclease noncatalytic subunit (ERCC1), an endonuclease noncatalytic subunit that is required for the repair of DNA lesions (Hsu *et al*., 2010); and interleukin‐8 (Hwang *et al*., 2011) to contribute to the mesenchymal phenotype. Moreover, Snail1 acts as an activator by interacting with CREB binding protein, which prevents repressor complex formation and remodels the tumor microenvironment (Hsu *et al*., 2014).

#### 1.1.2 Slug

Slug (also known as Snail2) belongs to the Snail superfamily of zinc finger transcriptional factors (Nieto, 2002). Slug interacts with the corepressor nuclear receptor coreceptor and recruits C‐terminal binding protein 1 (CtBP1) for repressing E‐cadherin and triggering EMT (Hajra *et al*., 2002; Molina‐Ortiz *et al*., 2012; Nieto, 2002). Slug also binds to E2‐box sequence of the target genes promoter (*BRCA2* and *VDR*) and recruits CtBP1 and HDAC1 to suppress the gene expression (Hemavathy *et al*., 2000; Molina‐Ortiz *et al*., 2012; Tripathi *et al*., 2005). Overexpression of VDR upregulates E‐cadherin, downregulates *SNAI1, TWIST1,* and *MMP9*, and reduces the ability to form mammospheres, an attribute of breast normal and cancer stem cells (CSCs; Larriba *et al*., 2016; Pervin *et al*., 2013). Degradation of Slug consequently enhances E‐cadherin expression and represses cancer cell invasion (Mittal *et al*., 2008; Shih and Yang, 2011; Wang *et al*., 2009).

#### 1.1.3 ZEB1

Zinc finger E‐box binding homeobox 1 (ZEB1) binds to E‐boxes and represses the expression of E‐cadherin to induce EMT (Eger *et al*., 2005; Spoelstra *et al*., 2006; Witta *et al*., 2006). ZEB1 can function as an activator by interacting with Smads, signaling mediators of the transforming growth factor beta (TGF‐β) pathway, and the transcriptional coactivator p300 (Pena *et al*., 2006; Postigo *et al*., 2003). The EMT‐inhibiting transcription factor ovo‐like zinc finger 2 restricts EMT by directly inhibiting EMT‐inducing factor ZEB1 and induces MET (Hong *et al*., 2015; Kitazawa *et al*., 2016; Roca *et al*., 2013; Watanabe *et al*., 2014). ZEB1 is indicated as a key factor for pancreatic cancer progression. Depletion of ZEB1 suppresses stemness and colonization capacity of tumor cells in Pdx1‐cre‐mediated activation of mutant Kras and p53 (KPC) model of pancreatic cancer. In this model, EMT‐TFs Snail1 and Twist1 had no such effect (Krebs *et al*., 2017; Zheng *et al*., 2015). Krebs *et al*. (2017) also suggested that there are considerable functional variabilities and tissue specificities among different EMT‐TFs. With regard to the interplay between ZEB1 and other EMT‐TFs, Snail1 acts cooperatively with Twist1 to control the expression of ZEB1 (Dave *et al*., 2011).

#### 1.1.4 ZEB2

Zinc finger E‐box binding homeobox 2 (ZEB2) acts as a transcriptional repressor and regulates downstream targets either dependent or independent of the CtBP1 corepressor complex (van Grunsven *et al*., 2003; Shi *et al*., 2003). ZEB2 induces EMT by binding to the E‐cadherin promoter and repressing the transcription of E‐cadherin (Comijn *et al*., 2001). Moreover, ZEB2 has been shown to repress the expression of several genes encoding junctional proteins, including desmosomal proteins desmoplakin and plakophilin 2 and tight junction protein claudin 4 (Vandewalle *et al*., 2005). ZEB2 is regulated by sumoylation, which attenuates gene repression by the disruption of CtBP1 recruitment (Long *et al*., 2005).

#### 1.1.5 Twist1

Twist1, a basic helix‐loop‐helix transcriptional factor, is a master regulator of gastrulation and mesoderm specification (Castanon and Baylies, 2002; Furlong *et al*., 2001) and is recently demonstrated to be essential to mediate cancer metastasis (Yang *et al*., 2004). Ectopic expression of Twist1 upregulates mesenchymal cell markers (fibronectin, vimentin, smooth muscle actin, and N‐cadherin) and a loss of epithelial markers (E‐cadherin, and α‐ and γ‐catenin), and induces EMT (Kang and Massague, 2004; Yang *et al*., 2004). Twist1 has been shown to play a vital role in the intravasation step of metastasis, angiogenesis, and chromosomal instability (Mironchik *et al*., 2005; Yang *et al*., 2004). Under hypoxic condition, a principal feature of malignancies, HIF‐1α promotes EMT through the induction of Twist1 (Yang *et al*., 2008). Twist1 in turn activates Bmi1, and both of them are essential for promoting EMT and tumor‐initiating capacity (Yang *et al*., 2008, 2010). A report by Tsai *et al*. (2012) also indicated that turning off Twist1 reversed the EMT process, leading to the subsequent occurrence of MET for colonization and the formation of metastases, indicating that Twist1 is an important regulator of epithelial plasticity during cancer metastasis.

### 1.2 Signaling pathways for EMT induction

EMT transcription factors can be activated through different pathways, which strongly suggest the convergence of diverse pathways on common targets during EMT (Lamouille *et al*., 2014). TGF‐β deposited in the surrounding stroma or secreted from tumor cells induces the expression of both ZEB1 and Snail1, thereby triggering EMT to promote tumor progression and metastasis (Korpal *et al*., 2008; Zavadil and Bottinger, 2005). Notch signaling pathway plays an important role in physiological and pathologic conditions through the induction of EMT (Niessen *et al*., 2008; Timmerman *et al*., 2004; Wang *et al*., 2010; Zavadil *et al*., 2004). WNT family proteins and growth factors that act through receptor tyrosine kinases have also been shown to induce EMT (Lamouille *et al*., 2014). Hypoxia induces the expression of Twist1 or Snail to promote EMT during cancer progression (Peinado and Cano, 2008).

### 1.3 miRNAs for regulation of EMT

miRNA that selectively target mRNA for the degradation of mRNA or translational repression also participate in the regulation of the EMT process (Ambros, 2004; Lamouille *et al*., 2013). For example, the miR‐200 family miRNAs have been shown to repress the expression of ZEB1 and ZEB2, thereby maintaining cancer cells in the epithelial phenotype (Gregory *et al*., 2008; Korpal *et al*., 2008; Park *et al*., 2008). ZEB1/2 and miR‐200 family members have a double‐negative feedback loop that controls the balance between epithelial and mesenchymal states (Bracken *et al*., 2008; Gregory *et al*., 2011). miR‐103/107 induces EMT by targeting Dicer, a key component of the miRNA processing machinery, to downregulate the level of miR‐200 in breast cancer cells (Martello *et al*., 2010). Inhibition of the let‐7d causes EMT (Huleihel *et al*., 2014; Pandit *et al*., 2010). miR‐181a mediates TGF‐β‐induced EMT (Brockhausen *et al*., 2015). miR‐9 directly targets the E‐cadherin‐encoding mRNA *CDH1*, leading to an EMT‐like conversion (Ma *et al*., 2010). In summary, signaling within the microenvironment triggers the activation of EMT‐TFs, resulting in the occurrence of EMT in cancer cells. miRNAs also function as major mediators of EMT by regulating the expression of EMT‐TFs.

## 2 EMT and cancer stemness

In the past decade, accumulating evidence has shown that EMT permits cancer cells to acquire stem cell properties for metastasis and dissemination. Here, we will focus on the association between EMT and cancer stemness.

### 2.1 Cancer stem cells

Intratumoral heterogeneity contributes to therapeutic resistance and results in disease recurrence (Hanahan and Weinberg, 2011). CSCs are a small population of cancer cells with the characteristics of self‐renewal, tumor initiation, and chemotherapy resistance (O'Brien *et al*., 2007; Ricci‐Vitiani *et al*., 2007; Todaro *et al*., 2007, 2014). The existence of CSCs was initially intensively debated; however, the concept of CSCs has been strongly supported by the application of spontaneous tumor mouse models and genetic tracing (Chen *et al*., 2012; Driessens *et al*., 2012; Schepers *et al*., 2012). Moreover, the term ‘stemness’, which was initially used to describe the properties of normal stem cells, has been expanded to illustrate the feature of CSCs with reference to the molecular signatures that control and maintain the stem cell state. In experimental models, stemness is generally defined as an increase in cancer type‐specific stem cell markers. The reported markers for CSCs in different types of cancers are illustrated in Table 1. Furthermore, serial replating of tumorspheres and *in vivo* serial repopulation assays have been applied as the standard procedures for testing the self‐renewal ability of cancer cells.

**Table 1:** CSC markers for different tumor types

Cancer types	CSC markers	Features/Reference
Breast	ALDH1	Tumor initiation in xenograft, poor prognostic factor, metastasis (Ginestier *et al*., 2007)
	CD44	Mammosphere formation, tumor initiation in xenograft, poor prognostic factor, metastasis (Al‐Hajj *et al*., 2003; Leth‐Larsen *et al*., 2012; Ponti *et al*., 2005)
	Sox2	Mammosphere formation, tumor initiation in xenograft (Leis *et al*., 2012)
Colon	LGR5	Increase pluripotency and self‐renewal (lineage tracing); induces clonogenicity and tumorigenicity (Barker *et al*., 2007; Kemper *et al*., 2012)
	CD24	Increase carcinogenesis; express in spheroid cultures (Sagiv *et al*., 2006; Vermeulen *et al*., 2008)
	CD29	Increase colony formation; express in spheroid cultures (Fujimoto *et al*., 2002; Vermeulen *et al*., 2008)
	CD44	Tumor initiation in xenograft, colony formation; poor prognostic factor, lymph node infiltration (Dalerba *et al*., 2007; Du *et al*., 2008; Huh *et al*., 2009)
	CD133	Tumor initiation in xenograft, sphere formation (Ricci‐Vitiani *et al*., 2007)
Head and neck	Oct4	Sphere formation, chemoresistance, invasion, migration, tumor initiation in xenograft, poor prognostic factor (Koo *et al*., 2015; Liao *et al*., 2016)
	CD44	Tumor initiation in xenograft, colony formation, sphere formation (Krishnamurthy *et al*., 2010; Prince *et al*., 2007)
	ALDH1	Tumor initiation in xenograft, colony formation, sphere formation, radioresistance (Krishnamurthy *et al*., 2010; Major *et al*., 2013)
Liver	CD133	Tumor initiation in xenograft, clonogenicity (Yin *et al*., 2007)
	SALL4	Poor prognostic factor, tumor proliferation, chemoresistance, tumor initiation in xenograft (Oikawa *et al*., 2013)
	ALDH1	Tumor initiation in xenograft, proliferation, sphere formation (Ma *et al*., 2008)
Pancreas	CD24/CD44/EpCAM	Tumor initiation in xenograft (Li *et al*., 2009)
	CD133	Metastasis, poor prognostic factor (Hermann *et al*., 2007; Li *et al*., 2015)
	CXCR4	Metastasis, poor prognostic factor (Hermann *et al*., 2007; Marechal *et al*., 2009; Wang *et al*., 2015)
Prostate	CD133	Proliferation, invasion, clonogenicity, glandular regeneration (Collins *et al*., 2005; Vander Griend *et al*., 2008)
	CD44	Tumor initiation in xenograft, proliferation, clonogenicity, metastasis, poor prognostic factor (Hurt *et al*., 2008; Li *et al*., 2007; Patrawala *et al*., 2006)
	EpCAM	Tumor initiation in xenograft, metastasis (Deng *et al*., 2015; Li *et al*., 2007)

### 2.2 Correlation between EMT and stemness

Exposing human mammary epithelial cells to TGF‐β or the ectopic expression of Snail1/Twist1 induces a cell population with stem cell characteristics, including enhanced expression of CD44 (CD44high) and low expression of CD24 (CD24low) and the ability to form mammospheres (Mani *et al*., 2008). Prostate cancer cells with the mesenchymal phenotype display stem‐like properties, including increased expression of the pluripotency genes Sox2, Nanog, and Oct4, enhanced clonogenic and sphere‐forming ability, and tumorigenicity *in vivo* (Kong *et al*., 2010). In pancreatic cancer, ZEB1 is the critical link between the activation of EMT and the acquisition of stem‐like properties and functions by suppressing miR‐200 family members, which are strong inducers of epithelial differentiation. Activation of ZEB1 promotes EMT and the expression of stem cell factors such as Sox2 and Klf4 (Wellner *et al*., 2009). Bmi1, a polycomb‐group protein that maintains self‐renewal, is directly regulated by Twist1, which links EMT to tumor‐initiating ability (Wu and Yang, 2011; Wu *et al*., 2012; Yang *et al*., 2010). The EMT process can also confer resistance to senescence. Twist1/2 and ZEB1/2 override oncogene‐induced premature senescence by inhibiting p53‐ and Rb‐dependent pathways (Ansieau *et al*., 2008; Morel *et al*., 2012; Ohashi *et al*., 2010). Furthermore, Twist1 acts together with Bmi1 to suppress the expression of let‐7, a microRNA expressed during stem cell differentiation, leading to cancer stemness (Yang *et al*., 2012). Downregulation of let‐7 activates the chromatin modifier ARID3B to promote expression of stemness genes through histone modification (Liao *et al*., 2016). In colon CSCs, Snail1 mediates the switch from asymmetric to symmetric cell division, indicating a role for EMT in increasing the size of the CSC pool (Hwang *et al*., 2014). Slug‐driven EMT program is important for inducing the entrance into adult stem cell state; however, it is not sufficient to induce this change in ‘differentiated’ luminal cells. Instead, activation of an additional genetic program through expression of Sox9 is required to work in concert with the EMT program to induce stem cells (Guo *et al*., 2012).

Intriguingly, EMT has also been shown to inhibit the development of stem‐like traits in certain studies (Celia‐Terrassa *et al*., 2012; Korpal *et al*., 2011; Sarrio *et al*., 2012), a finding that contradicts the concept of EMT‐induced stemness. Further evidence has shown that, in human breast cancer cells, knockdown of paired‐related homeobox transcription factor 1 (Prrx1), a recently identified EMT inducer, increased mammosphere formation, self‐renewal capacity, and the proportion of enhanced expression of CD44 (CD44high) and low expression of CD24 (CD24low) CSCs (Ocana *et al*., 2012). Moreover, another study showed that Twist1 is essential for the acquisition of CSC properties; however, cancer stemness is independent of EMT or tumor invasion, implying that EMT and stemness are regulated separately (Beck *et al*., 2015). Transient activation of Twist1 promotes cancer stemness, even when EMT has not been induced (Schmidt *et al*., 2015). Taken together, this indicates that EMT is closely associated with but is not necessary for cancer stemness. EMT‐TFs are the critical mediators that link EMT to stemness, but the mechanisms are different, including epigenetic and miRNA regulation; in other words, the regulation of EMT and stemness are an independent function of the same EMT‐TFs. This correlation between EMT and cancer stemness is more complicated than expected and deserves intensive investigation in the future.

## 3 Cell plasticity and cancer stemness

Studies in induced pluripotent stem cells (iPSCs) showed that MET, the reverse process of EMT, is a prerequisite for the reprogramming of fibroblasts to iPSCs (Li *et al*., 2010; Samavarchi‐Tehrani *et al*., 2010). During the reprogramming process, Oct4/Sox2 represses the expression of Snail1, c‐Myc reduces the expression of TGF‐β1 and TGF‐β receptor П, and Klf4 activates the expression of E‐cadherin. All these events result in MET (Li *et al*., 2010). During tumor progression, MET is considered an essential process for metastatic colonization (Nieto, 2013). Evidence of EMT in clinical specimen is the fact that the histology of metastatic tumors exhibits the epithelial phenotype rather than the mesenchymal‐like phenotype, suggesting that the reversion of EMT occurs during metastatic colonization (Yao *et al*., 2011). Moreover, miR‐200 family miRNAs were shown to promote MET, which was also found to increase metastatic colonization (Dykxhoorn *et al*., 2009; Perdigao‐Henriques *et al*., 2016). In addition to metastatic colonization, MET has also been noted to promote the stemness of cancer cells. For example, inhibitor of differentiation 1 (Id1) induces MET and the stem‐like phenotype by antagonizing Twist1 (Stankic *et al*., 2013). Connective tissue growth factor has been noted to enhance stem‐like properties and trigger MET in head and neck cancer cells (Chang *et al*., 2013). Furthermore, transient expression of Twist1 induces long‐term invasiveness and colonization capability by promoting the coexistence of the features of epithelial and mesenchymal cells (Schmidt *et al*., 2015). This result suggests that an ‘intermediate state’ of cancer cells may be more flexible in terms of cell invasion and the regulation of stem‐like properties.

**Figure 2:**
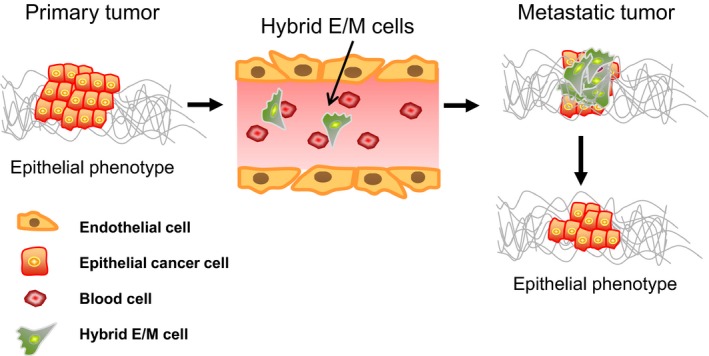
A model for depicting cellular plasticity for cancer metastasis. In primary tumors, most cancer cells have an epithelial type. In metastatic cancer, hybrid epithelial/mesenchymal (E/M) cells or partial EMT is favorable for cancer dissemination. When the hybrid E/M cells reach the metastatic site, they will revert back to epithelial cells to form metastatic colonies, possibly via rapid kinetics. Therefore, the epithelial/mesenchymal features and stem‐like properties are no longer a fixed state. A dynamic or a flexible feature of E/M phenotype is a better description for the plasticity of cancer cells.

A concern of previous studies is that most instances of EMT or MET were achieved by the forced expression of certain factors, which fixed cells in a terminal epithelial or mesenchymal state and may not reflect the dynamic process of transition between epithelial and mesenchymal status *in vivo*. For example, circulating tumor cells (CTCs) have been shown to express both epithelial and mesenchymal markers (Bonnomet *et al*., 2012; Lecharpentier *et al*., 2011; Paterlini‐Brechot and Benali, 2007; Raimondi *et al*., 2011; Yu *et al*., 2013). In patients with advanced metastatic cancer, a high frequency of ‘hybrid’ CTC populations expresses CSC markers (Armstrong *et al*., 2011; Theodoropoulos *et al*., 2010). A recent study that used intravital microscopy to observe epithelial‐mesenchymal plasticity without artificially modifying the expression of EMT regulators showed that epithelial‐mesenchymal plasticity occurs during the migration process but not when cells enter the circulation. This study also observed that mesenchymal cells adopt the epithelial state after several rounds of cell division upon reaching metastatic sites (Beerling *et al*., 2016). Furthermore, the hybrid epithelial/mesenchymal (E/M) cells in primary ovarian cancer cells and prostate cancer cells showed higher self‐renewal and tumor‐initiating ability (Ruscetti *et al*., 2015; Strauss *et al*., 2011). The concept of hybrid E/M cells in metastatic colonization is shown in Fig. 2. Therefore, stemness properties are no longer a feature of a fixed state, but follow the changes in the cells as a flexible feature. Further studies are necessary to clarify the mechanism and significance of epithelial plasticity and stemness in tumor cells.

## 4 Conclusions

Experimental models of EMT have been used for decades and have established a foundation for us to elucidate the mechanisms underlying EMT, metastasis, and tumor initiation. However, this dichotomy between the epithelial and the mesenchymal states may be oversimplified and may not precisely reflect the situation *in vivo*. The concept of an ‘intermediate‐state’, or so‐called partial EMT, provides a possible explanation for this controversy. The phenomenon of partial EMT has been found to occur during the process of embryo development and in wound healing, and a growing body of evidence indicates the existence of partial EMT in cancer biology. Hence, the development of an *in vivo* model will be important for providing a research tool for us to use in elucidating the dynamic changes in the epithelial‐mesenchymal phenotype and the regulation of stemness properties in pathophysiological microenvironments. Considering a process of plastic change between the epithelial and the mesenchymal states is more useful than considering the process of a fixed transition for our understanding of cancer progression and metastasis.
